# TSS seq based core promoter architecture in blood feeding Tsetse fly (*Glossina morsitans morsitans*) vector of Trypanosomiasis

**DOI:** 10.1186/s12864-015-1921-6

**Published:** 2015-09-22

**Authors:** Sarah Mwangi, Geoffrey Attardo, Yutaka Suzuki, Serap Aksoy, Alan Christoffels

**Affiliations:** South African MRC Bioinformatics Unit, South African National Bioinformatics Institute, University of the Western Cape, Bellville, South Africa; Department of Epidemiology of Microbial Diseases, Yale School of Public Health, New Haven, CT 06510 USA; Department of Medical Genome Sciences, University of Tokyo, Tokyo, Japan

**Keywords:** *Glossina morsitans morsitans*, Transcription start site, Core promoters, TSS seq

## Abstract

**Background:**

Transcription initiation regulation is mediated by sequence-specific interactions between DNA-binding proteins (transcription factors) and cis-elements, where BRE, TATA, INR, DPE and MTE motifs constitute canonical core motifs for basal transcription initiation of genes. Accurate identification of transcription start site (TSS) and their corresponding promoter regions is critical for delineation of these motifs. To this end, the genome scale analysis of core promoter architecture in insects has been confined to *Drosophila*. The recently sequenced Tsetse fly genome provides a unique opportunity to analyze transcription initiation regulation machinery in blood-feeding insects.

**Results:**

A computational method for identification of TSS in newly sequenced Tsetse fly genome was evaluated, using TSS seq tags sampled from two developmental stages namely; larvae and pupae. There were 3134 tag clusters among which 45.4 % (1424) of the tag clusters mapped to first coding exons or their proximal predicted 5′UTR regions and 1.0 % (31) tag clusters mapping to transposons, within a threshold of 100 tags per cluster. These 1393 non transposon-derived core promoters had propensity for AT nucleotides. The −1/+1 and 1/+1 positions in *D. melanogaster*, and *G. m. morsitans* had propensity for CA and AA dinucleotide*s* respectively. The 1393 tag clusters comprised narrow promoters (5 %), broad with peak promoters (23 %) and broad without peak promoters (72 %). Two-way motif co-occurrence analysis showed that the MTE-DPE pair is over-represented in broad core promoters. The frequently occurring triplet motifs in all promoter classes are the INR-MTE-DPE, TATA-MTE-DPE and TATA-INR-DPE. Promoters without the TATA motif had higher frequency of the MTE and INR motifs than those observed in *Drosophila,* where the DPE motif occur more frequently in promoters without TATA motif. Gene ontology terms associated with developmental processes were overrepresented in the narrow and broad with peak promoters.

**Conclusions:**

The study has identified different motif combinations associated with broad promoters in a blood-feeding insect. In the case of TATA-less core promoters, *G.m. morsitans* uses the MTE to compensate for the lack of a TATA motif. The increasing availability of TSS seq data allows for revision of existing gene annotation datasets with the potential of identifying new transcriptional units.

**Electronic supplementary material:**

The online version of this article (doi:10.1186/s12864-015-1921-6) contains supplementary material, which is available to authorized users.

## Background

Tsetse flies (*Glossina* spp) are the biological vectors for Trypanosomes, the causative agents of Human African Trypanosomiasis (HAT). HAT is a debilitating disease that continues to present a major public health problem and a key factor limiting rural development in vast regions of tropical Africa. To augment the current vector control efforts, the tsetse fly (*Glossina morsitans morsitans (G. m. morsitans)*) genome was recently sequenced [1]. To elucidate the organization of the basal transcription initiation machinery and thereby improve genome annotation, *G. m. morsitans* core promoters and their corresponding transcription factor binding sites were analyzed in this study.

The core promoter constitutes the minimal portion of the promoter required to properly initiate transcription. It encompasses transcription start site (TSS) extending either upstream or downstream for ~50 bases [2–4]. The TSS can be defined as the first nucleotide copied at 5′ end of corresponding mRNA. For eukaryotic protein coding genes, transcription initiation is facilitated by RNA polymerase II in co-operation with other transcription factors (TFs) that bind transcription factor binding sites (TFBSs). The core promoter architecture has been elucidated for human and mouse [5] and the fruit fly [6–10] among which canonical core promoter motifs are conserved [2, 4, 11]. Canonical core promoter motifs include TATA, the initiator (INR), TFIIB recognition element (BRE) and downstream promoter element (DPE) motifs.

Recent developments in high-throughput next generation sequencing (NGS) technologies have been employed to facilitate the elucidation of transcriptional control mechanisms through promoter identification and expression profiling. For example, TSS seq [12], a recently developed NGS technique, employs oligo-capping technology [13] to capture 5′ end of mRNAs, providing information on location of TSS and permitting analysis of high-throughput transcript expression profiles. Since outputs of TSS seq experiment are tags exclusively enriched with 5′ end of transcripts, mapping TSS seq tags on a genome appears as a peak signal.

High-throughput methods for TSS detection on a genome-wide scale have revealed that metazoan transcription initiates across genomic windows of varying lengths [14]. This variation in initiation patterns led to two major promoter classification schemes namely; (i) narrow promoters, whose transcription initiation proceeds from a single nucleotide or within a region of several nucleotides, and (ii) broad promoters where transcription initiates in a region of 100–200 bases reviewed by Lenhard et al. [14]. In *Drosophila melanogaster*, narrow promoters mainly harbor the TATA and INR motifs while broad promoters tend to harbor variably located core promoter motifs [8, 15].

Computational prediction coupled with experimental validation has helped identify an ever-increasing number of core promoter elements such as motif ten element (MTE) [16]. This association of discrete core promoter motifs with either of the promoter classes has highlighted the importance of core element diversity for transcription regulation [8]. Vertebrate promoters have normally been associated with presence of CpG island with, half of protein coding genes harbored in CpG islands in human genome [17, 18]. The case is different for insects where promoters are more A and T rich as observed in *Drosophila* [19], suggesting fundamental difference in global promoter architecture between mammals and insects.

Genome wide analysis of core promoter architecture is poorly understood, despite advances in deciphered motif profiling of specific genes in genomes of blood feeding insects such as mosquito [20–23]. *G. m. morsitans* promoters present a valuable tool both for studying vector parasite interactions and development of novel Trypanosomiasis control strategies such as Trypanosome-refractory Tsetse flies through expression of anti-Trypanosome genes. With the completion of the *G. m. morsitans* genome [1], this study endeavors to gain insight into core promoter architecture of a blood feeding insect. Approximately six million TSS seq reads sampled from the larval and pupal developmental stages of *G. m. morsitans* were used to identify the TSS of corresponding genes. Regions flanking the TSSs were used to assess the core promoter architecture in *G. m. morsitans*.

## Results

### Genome mapping and TSS seq clustering

The current assembly of *G. m. morsitans* genome consists of 13,807 scaffolds of which 3058 contain at least one gene [1]. TSS seq reads mapped onto 2896 of the 3058 gene-containing scaffolds and 2736 out of 10,749 scaffolds without genes. TSS seq reads with at least one overlapping read were grouped into clusters known as tag clusters. Tag clusters containing at least 100 TSS seq reads were deemed to have a strong transcriptional signal and were selected for downstream analysis. Most tag clusters mapped onto gene-containing scaffolds (Table 1). The scaffolds without genes may be intergenic regions, suggesting unassembled regions of the current *G. m. morsitans* genome. Overall, 3134 tag clusters were obtained with a cluster defined by at least 100 reads per cluster (Table 2). About 65 % (2033/3134) tag clusters mapped onto annotated genes with the rest mapping onto intergenic regions, probably corresponding to previously unannotated transcripts or non-coding RNA TSS. Most (70 %) of the tag clusters mapping onto annotated genes were located on the first coding exons or their proximal 5′UTR regions. An additional 87 tag clusters mapped onto the gene-less scaffolds that probably represent intergenic regions. The connotation ‘intergenic’ for gene-less scaffolds is used with caution because of the fragmented nature of the genome coupled with irregular gene distributions. About two-thirds of gene-containing scaffolds harbor only one gene, with median gene length of about 4488 base pairs. Tag clusters located within 4488 base pairs from either end of the scaffold, were classified as “periphery tag clusters” and could not be associated with any gene.

**Table 1:** Summary of TSS seq genome mapping statistics

Parameter	Count
Gene-containing scaffolds	3,058
Gene-containing scaffolds with TSS seq reads	2,896 (6,513,739^a^)
Gene-less scaffolds	10,749
Gene-less scaffolds with TSS seq reads	2,736 (108,685^a^)

^a^Absolute numbers of reads mapping (97 % of the TSS seq reads mapped onto gene-containing scaffolds)

**Table 2:** Summary of clustering statistics

Parameter	Number of clusters
Number of clusters with > = 100 TSS seq reads	3134
Total number of tag clusters in genic region	2033
Total number of tag clusters in CDS1^a1^ and 5UTR	1424
Total number of tag clusters in other genic regions	609
Total number of tag clusters outside genic regions	1014
Tag clusters in gene-less containing scaffolds	87

^a^CDS1 = First coding exon

### Core promoter extraction

Core promoters were defined as 100 nucleotides (−50/+50) TSS surrounding the TSS. By setting the threshold at 100 tags per cluster, 1424 tag clusters were located on the first coding exons and their proximal 5′UTR regions. 197 genes had more than one candidate tag cluster. In such a case, the cluster with more tags was selected for further analysis. Their corresponding core promoters were extracted. Approximately 31 core promoters were entangled with transposons and were therefore excluded from further analysis, effectively reducing core promoter set used for motif assignment to 1393.

### Delineation of promoter classes and distribution of their core nucleotides

**Fig. 1:**
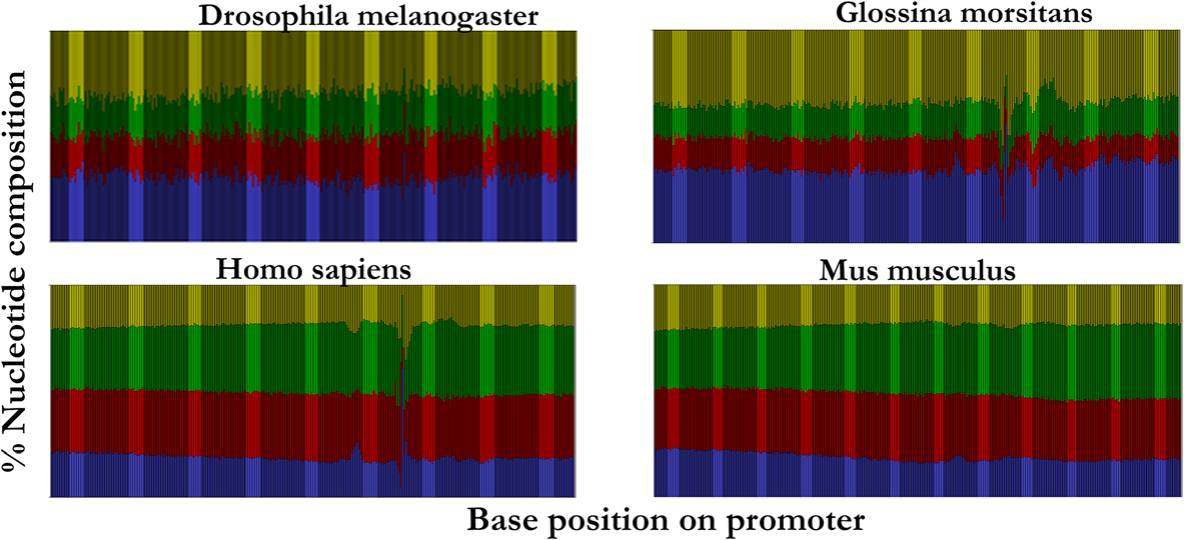
Combined nucleotide composition graphs for *D.melanogaster*, *Glossina morsitans, H. sapiens* and *M. musculus*. For each chart, the x-axis represents 300 (−200 to +100) bases around the TSS while the y-axis represents % base composition at each nucleotide position. Base colors are represented as follows; Red = C, Blue = A, Green = G, Yellow = T

The peakedness (Sg value) of a tag cluster decreased with increasing tag cluster size. Clusters with single TSS had *Sg* value of 1 while those with the highest number of TSS (220) had an Sg value of 0.0023. Essentially higher *Sg* values represent peaked distributions and vice versa. At least 70 % of tag clusters had no peak, as defined by a cut off of at least 50 % of the total reads in the mode position for a tag cluster (Table 3). Alignment of −200/+100 regions surrounding the TSS revealed clear distinction in nucleotide composition between mammalian and insect promoters (Fig. 1). Insect promoters exhibit propensity for the AT dinucleotide whilst mammalian promoters exhibit propensity for the CG dinucleotides.

**Table 3:** Summary of tag cluster types

Tag cluster type	Absolute number	% Of total
Narrow	69	5
Broad with peak	314	23
Broad without peak	1010	72

**Fig. 2:**
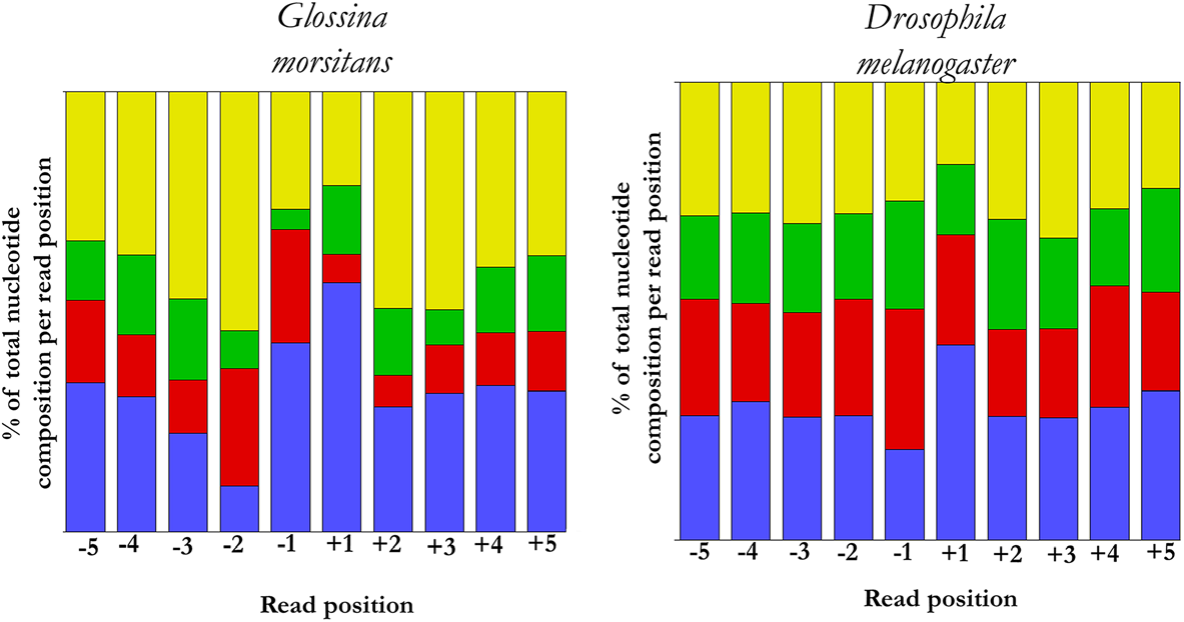
Nucleotide frequency distribution surrounding the TSS. The y-axis represents % base composition at each nucleotide position. The x-axis represents 10 nucleotides surrounding the TSS. Red = C, Blue = A, Green = G, Yellow = T. *Glossina morsitans* core promoter*:* the −1/+1 positions shows propensity for AA dinucleotide while *Drosophila melanogaster* core promoters: the −1/+1 positions shows propensity for CA dinucleotide

The INR motif, encompassing TSS, varies substantially between studies ranging from a TCA (G/T) TC(C/T) to a single dinucleotide (pyrimidine (C/T)–purine (A/G)) [14]. Notably, majority of promoters only have one or a few of these patterns, and some patterns are typically found in certain species. The −5/+5 region surrounding the TSS was extracted for a closer scrutiny of the INR in *G. m. morsitans* and *D melanogaster* core promoters (Fig. 2). The nucleotide frequency distribution show that in *G. m. morsitans* core promoters, the −1/+1 positions shows propensity for AA dinucleotide while the *D melanogaster* core promoters positions −1/+1 show propensity for the CA dinucleotide. The base composition around *G. m. morsitans* INR sequence suggests that this analysis assigned the TSS properly for most of *G. m. morsitans* core promoters.

### Annotation of core promoter motifs

#### Real vs. random motifs

Every motif finding algorithm is ordinarily exposed to spurious matches that may appear as significant as the ones in question [24] and the number of core promoter motifs in the random dataset in Table 4 confirms this. By knowing the biologically functional genomic windows of these motifs a priori, motifs that did not occur in biologically functional genomic windows were omitted, partly correcting for false matches. The number of motifs identified within the narrow (P < =0.00164), broad with dominant peak (P < =0.00135) and broad without dominant peak (P < =0.00185) promoter datasets exceeded those identified in a randomized dataset (Table 4). There is a clear enrichment for all but the BREu motif between the real and random datasets across all promoter classes.

**Table 4:** Comparison of core promoter motifs instances between true and random datasets

Core promoter classes	BREu	BREd	TATA	INR	MTE	DPE
Narrow (true)	15	13	27	28	16	20
Narrow (random)	9	5	10	10	13	5
Broad with peak (true)	38	61	88	87	74	80
Broad with peak (random)	32	33	45	42	39	47
Broad without peak (true)	129	180	163	181	190	166
Broad without peak (random)	103	106	131	133	102	126

### Promoter motif profiles in narrow and broad classes

**Fig. 3:**
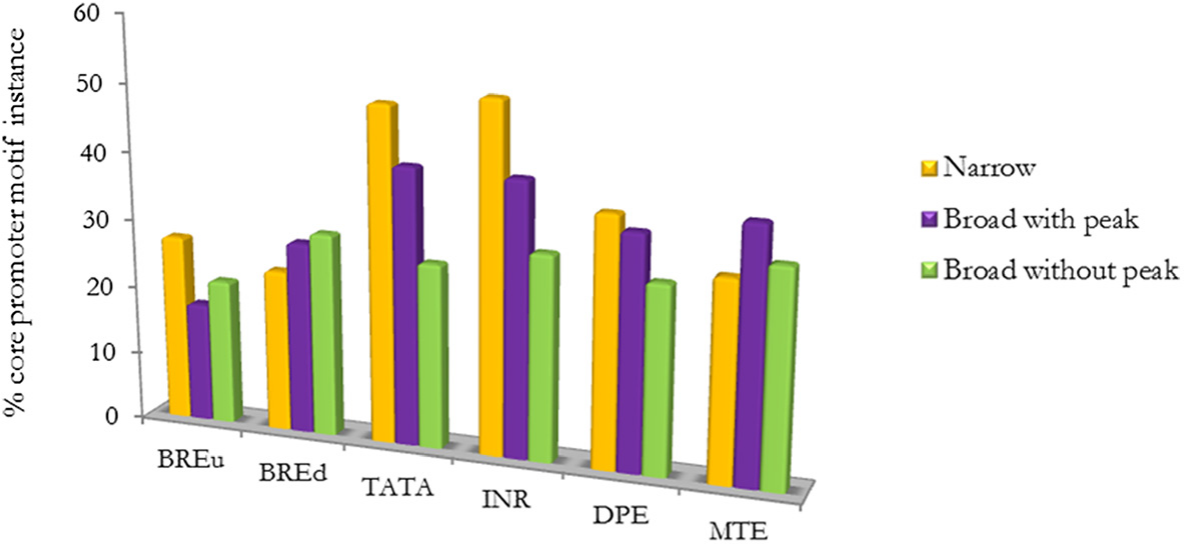
Distribution of core promoter instances in the various promoter classes. The figure depicts a clear separation in core motif frequency between narrow and broad promoters. The BREu motif is underrepresented in both broad core promoter categories

Canonical core promoter motifs were found in 74 % of *G. m. morsitans* core promoters. Overall, the INR, MTE and TATA motifs were present in 34 %, 33 % and 30 % of these core promoters respectively. The BREd motif was present in 29 % while the BREu motif was present in 21 % of the core promoters (Table 5). There was a clear separation in core motif frequency between the three core promoter categories (Fig. 3). The BREu motif is underrepresented in both broad core promoter categories (Fig. 3). Approximately 50 % of the narrow core promoters harbored the TATA and INR motifs. This suggests that the INR may be of equal importance to transcription for narrow promoters as the TATA motif in *G. m. morsitans*. Narrow core promoters with focused initiation are associated with TATA and INR motifs [7].

**Table 5:** Percent core promoter motifs occurrence

Motif	% Occurrence
BREu	21
BREd	29
TATA	32
INR	34
DPE	30
MTE	33

**Fig. 4:**
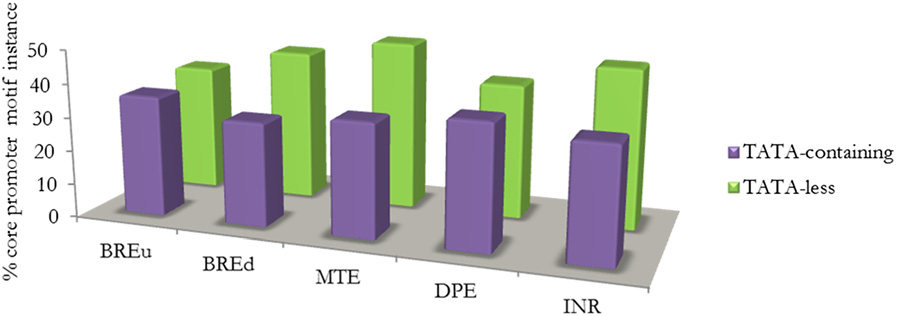
Distribution of core promoter motifs in the TATA containing and TATA-less core promoters. There was no significant difference in the frequency of the BREu motif, however, the MTE and INR motifs exhibit higher frequency in TATA-less promoters

About 23 % of *G. m. morsitans* core promoters had TATA motif. There was no significant difference in the frequency of the BREu motif, however, the remaining core promoter motifs had a higher frequency in TATA-less promoters, notably the MTE and INR motifs (Fig. 4). The DPE was previously reported to occur frequently in TATA-less promoters [25], but these results show that the INR and MTE are more frequent in *G. m. morsitans* TATA-less promoters. Indeed Lim and colleagues [16] showed that the MTE in the absence of a DPE can compensate for the loss of a TATA motif.

### Motif co-occurrences in narrow vs. broad core promoters

For two-way motif combinations, the 95 % confidence interval calculations indicated that the MTE-DPE pair (22 %) is significantly over-represented within the broad without peak core promoter category. The BREu–MTE and BREd-DPE pairs are under-represented within the narrow promoter category, and BREu-DPE and BREd-DPE are under-represented within the broad with peak core promoter category indicating possible non-cooperativity in binding. All other motif pairs fell outside the 95 % confidence interval. Nevertheless the higher frequency of co-occurrence for the INR-MTE and MTE-DPE (Broad with peak promoters), and TATA-INR and TATA-DPE pairs (narrow promoters) points to possible cooperativity during transcription activation. The TATA-INR and MTE-DPE cooperation has been observed in mammalian and *Drosophila* promoters [26, 27] (Table 6).

**Table 6:** Two-way motif co-occurrences

	% Co-occurrence in core promoter category		
Motif combination	Narrow	Broad with peak	Broad without peak
BREu-TATA	14	8	13
BREu-BREd	12	13	15
BREu-INR	19	13	10
BREu-MTE^b^	7	12	10
BREu-DPE^c^	17	11	7
TATA-BREd	21	13	11
TATA-INR	28	14	10
TATA-MTE	23	13	9
TATA-DPE	27	13	13
BREd-INR	11	13	12
BREd-MTE	12	13	11
BREd-DPE^b,c^	10	9	11
INR-MTE	22	20	13
INR-DPE	14	17	12
MTE-DPE	20	20	22^a^

^a^Statistically over represented in the broad without peak promoter category

^b^Statistically under represented in the narrow promoter category

^c^Statistically under represented in the broad without peak promoter category

For three-way core promoter motif combinations, there was no statistically over-represented trio within each promoter category. However, the top two trios occurring in each promoter category indicate a possible synergistic role. For example, Lim and colleagues [16] demonstrated that the MTE motif could function independently of, or synergistically with, both TATA and DPE motifs. The BREu-BREd -MTE pair was under-represented across all core promoter categories indicating that the trio may not act in synergy for all promoter classes (Table 7). Other trios were either under represented in broad without peak category, broad with peak category or combination of the two (Table 7).

**Table 7:** Three-way motif co-occurrences

	% Co-occurrence in core promoter		
Motif combination	Narrow	Broad with peak	Broad without peak
BREu-TATA-BREd^a^	26	23	27
BREu-TATA-INR^c^	42	24	22
BREu-TATA-MTE^a^	35	24	25
BREu-TATA-DPE^a^	31	22	21
BREu-BREd-INR^a^	40	24	22
BREu-BREd-MTE^d^	22	24	20
BREu-BREd-DPE^a^	24	17	21
BREu-INR-MTE	35	27	21
BREu-INR-DPE^b^	35	29	19
BREu-MTE-DPE	28	31	22
TATA-BREd-INR	40	30	22
TATA-BREd-MTE	35	27	23
TATA-BREd-DPE	33	30	22
TATA-INR-MTE	26	36	25
TATA-INR-DPE	44	36	23
TATA-MTE-DPE	49	35	28
BREd-INR-MTE	20	31	23
BREd-INR-DPE	27	30	21
BREd-MTE-DPE	32	30	24
INR-MTE-DPE	40	32	29

^a^Statistically under represented only in with broad with peak core promoters

^b^Statistically under represented only in broad without peak promoters

^c^Statistically under represented in both broad promoter categories

^d^Statistically under represented in all promoter categories

### Gene ontology associations with various promoter classes

Ontology terms were assigned to 307 out of 1393 genes that represented different promoter classes. Selection of ontology terms in the 75th percentile of each promoter category showed that ontologies associated with developmental processes such as “structural constituents of cuticle” are over represented in narrow and broad with peak promoter classes (Additional files 1 and 2). Cuticular constituents are involved in chitin metabolism and molting and are crucial to insect growth and morphogenesis.

Certain ontologies were represented in specific promoter classes. ATP binding and DNA binding were over-represented in the narrow promoters (Additional file 1). Signaling pathways such as a small GTPase mediated signal transduction was over-represented in broad without peak promoters (Additional file 3).

## Discussion

The species-specific differences associated with nucleotide sequence composition informs the parameters used to develop ab initio gene prediction algorithms. For example, Lenhard and colleagues [14] demonstrated differences in promoter landscape among metazoan species. These comparisons tend to utilize the *Drosophila* genomic data when compared to mammalian species due to the high quality genomic and transcriptomic resources for *Drosophila*. The recent annotation of the second blood feeding disease vector, the tsetse fly, relied on gene prediction algorithms well suited to the *Drosophila* genome annotation project. Access to TSS seq data, albeit from two developmental stages only, provided the opportunity to investigate tsetse fly promoter composition in an attempt to improve genome annotation within this species and in preparation for the pending sequencing data of four related tsetse species. To this end, TSS seq data was generated for larvae and pupae developmental stages and mapped to the tsetse genome (version GmorY1) to characterize the promoters and delineate the primary TSS location.

### Core promoter organization in *G. m. morsitans*

In this study, approximately 1300 core promoters were extracted from the recently assembled Tsetse genome. Most of tag clusters (70 %) were identified in the 5′UTR and the first coding exon. Carninci and colleagues [28] used the variations in tag cluster location and composition to define promoter types. We applied this classification scheme to Tsetse and found that 95 % of the core promoters in this dataset are of the broad type. Within the broad category 76 % do not have preference for one initiation site. These are known as “broad without peak promoters”. The remainder (24 %) of promoters in the broad category have a preference for one initiation site. Narrow core promoters, which initiate over a narrow range of nucleotides, constitute a very small proportion of the total promoter count (6 %). This distribution is concordant with the picture that is emerging for metazoan transcriptional programs whereby few of the core promoters fit the “traditional” model of transcriptional regulation, that is, the narrow category. Majority of metazoan genes’ core promoters are of the broad type. Broader TSS initiation patterns may in theory be a consequence of non-specificity in the basal transcription machinery, and biological effects of such alterations on transcription are yet to be elucidated [8]. It was presumed that the combinatorial interaction of multiple TFs with the gene promoter is sufficient to explain the process of transcription. However, recent studies provided results to show that most eukaryotic genes possess multiple TSSs and by extension multiple promoters. These multiple promoters drive gene expression in a context-specific manner [28]. Possession of multiple promoters by extension generates diversity and complexity in the eukaryotic transcriptome.

### Tsetse fly core promoter’s propensity for AT nucleotides

The nucleotide frequency and distribution in Tsetse fly core promoters are similar to that of *D. melanogaster* [19] where the core promoters are characterized by propensity for the AT nucleotides. In *D. melanogaster*, AT enrichment peaks at approximately −200 bp from the TSS and microarray analysis showed these regions as nucleosome free mostly for active genes in *D. melanogaster* [29] and *Saccharomyces cerevisae* [30]. The positioning of nucleosomes along chromatin has been implicated in eukaryotic gene expression regulation because packaging of DNA into nucleosomes affects sequence accessibility. On the other hand, mammalian promoters depict propensity for the CG nucleotides. Vertebrate promoters have generally been associated with presence of CpG island, for instance, in the human genome, half of protein coding genes harbor CpG islands [17, 18]. The difference in nucleotide preference suggests a fundamental difference in global promoter architecture between mammals and insects. Perhaps, other mechanisms may perform the role of CpG islands in *G. m. morsitans* and *D. melanogaster*. These mechanisms have yet to be elucidated. While profiling ascidian promoters, Okamura and colleagues [31] postulated that CpG islands are not sufficiently ancient to be found in invertebrates and that these islands may have appeared early in vertebrate evolution through some active mechanism. The islands may have since been retained as part of vertebrate promoters. Indeed, introducing an artificial CpG island into mouse cells led to establishment of epigenetic patterns typical of promoter suggesting that mammalian CpG islands might be primed to be promoters by default [14, 32, 33].

### Variation of known core promoter motifs

To further validate reliability of our TSSs identification method, the presence of canonical core promoter motifs was examined. Variations in motif frequencies in narrow and broad promoters were investigated. Narrow promoters are characterized by only one or a few consecutive TSSs and are associated with genes that are expressed in tissue-specific manner. These promoters are enriched for the TATA motif. Broad promoters contain several TSSs over a large genomic window (usually not greater than 100 bp). In mammals, they are CpG rich and are usually found in constitutively expressed genes (review by Lenhard et al. [14]). In this study, high frequency of the TATA and INR (49 % and 51 % respectively) motifs was observed in narrow promoters. A similar pattern is observed for the broad with peak category (41 % and 40 % respectively) (Additional file 4). Since both the narrow and broad with peak classifications harbor a single dominant peak, these core promoter patterns may indicate the specificity of the transcription initiation machinery for peaked promoters. Despite its conservation in all eukaryotes, comprehensive analyses of *Drosophila* core promoters as well as mammals have suggested that the TATA occurs in approximately 10–30 % of all genes within a genome [6, 7, 19, 34, 35]. In this study, 23 % of *G. m. morsitans* core promoters harbored a TATA. Apart from the BREu motif, TATA-less promoters record a higher frequency of all other core promoter motifs. A similar observation was made by Gershenzon and colleagues [36] where they postulate that other core promoter motifs may provide a binding site for the basal transcription machinery in the absence of a TATA to mediate transcription. Indeed, the DPE was discovered through the analysis of the binding of purified TFIID to TATA-less genes [25]. Nucleotide conservation and core promoter motif’s distribution among *G. m. morsitans* and *D. melanogaster* genes indicate conservation of core promoter machinery among these insects.

### Variation of core promoter motifs co-occurrence

Most core promoters have at least one core-promoter motif at a functional position working as anchors for the basal transcription initiation machinery. However, the presence of a synergetic combination of two core promoter elements is often considerably stronger than a single element as it dictates the position of TSS. It is extremely rare for all motifs to be present in any given core promoter. Analysis of *G. m. morsitans* core promoter’s two-way motif co-occurrence revealed that the TATA-INR pair has the highest frequency among narrow core promoters whilst the MTE-DPE pair has the highest frequency for broad core promoters. Since high frequency of co-occurrence may indicate that the motifs exert their functions cooperatively, we postulate that the corresponding TFs for the TATA and INR as well as MTE and DPE exhibit synergistic interactions during transcription initiation for narrow and broad core promoters respectively. Indeed, the TATA-INR and MTE-DPE co-operation has been reported by other studies such as [16, 26, 27, 37]. In the broad without peak category, the highest frequency of three-way motif co-occurrence was found to be the INR-MTE-DPE triplet. Most studies on core promoter motifs have shown that neither the DPE nor the MTE exhibits core promoter activity in the absence of an INR [38]. Furthermore, our analysis of TATA-less core promoters shows propensity for the INR and MTE motifs and they have also been shown to compensate for the lack of a TATA motif [16, 39]. Intriguingly, this triplet has anchor points downstream of the TSS, and within the TSS itself. Thus, from a structural point of view this combination may mediate basal transcription initiation without necessarily positioning the RNA polymerase II complex very efficiently. The broad with peak category had the TATA-INR-DPE and TATA-INR-MTE combinations as the most frequent trios. The TATA-INR-MTE combination was also observed by Lim and colleagues [16]. Structurally, these triplets have anchor points on both sides of the TSS and within the TSS itself and may therefore position the RNA polymerase II complex efficiently. TATA-MTE-DPE combination was most frequent in the narrow core promoter’s category. The MTE exhibits synergy with the TATA and DPE motifs according to Gershon and colleagues [40]. Notably, 26 % of the core promoters lack known core promoter motifs, an observation that has been made in other studies [39–41]. It is hypothesized that undiscovered core promoter motifs might exist. However, the current ones are deemed sufficient to explain the RNA pol II mediated basal transcription initiation program for majority of genes.

### Repeat-recruited core promoters

Approximately one third of the *G. m. morsitans* genome is riddled with repeat motifs and 31 out of 1424 core promoters in this dataset were located on transposons. It has been recently discovered that retrotransposons and repeat elements are recruited as promoters and there is growing interest in the role of repeat elements in gene regulation. Indeed laboratory investigations have confirmed many specific examples of mammalian genes regulated by promoters donated by endogenous transposable motifs. For example, while using reporter constructs for Ewing cell lines, Guillon and colleagues [42] showed that transcription activation is highly dependent upon the number of repeats that are included in the construct. They postulated that microsatellites in promoters contribute to long-distance transcription regulation. In their review of metazoan promoters, Lenhard and colleagues [14] attribute approximately 200,000 human promoters to be retrotransposons-driven. In addition they state that these repeat driven promoters do not so far fit clearly into one of the main promoter classes namely, narrow and broad. According to Cohen and colleagues [43], repeat-recruited promoters have preference for tissue specific activity. A recent study by Lee and Maheshri [44] has shown the indirect impact on gene expression if the repetitive regions contain TFBSs which include transcription factor sequestration, aberrant activation of genes outside given promoter contexts and negative cooperativity in transcription factors. These events culminate in qualitative changes in the behavior of gene regulatory networks in which target genes are embedded. Vinces and colleagues [45], showed that in *S. cerevisae*, as many as 25 % of all gene promoters contain tandem repeat sequences and these genes driven by repeat-containing promoters show significantly higher rates of transcriptional divergence where variations in repeat length result in fluctuations in expression and local nucleosome positioning. This observation could be used in follow-up studies towards understanding of the effect of these tandem repeats on transcription control in upcoming *Glossina* genomes.

### Putative non-coding RNA transcription start sites

Non-coding RNA genes include highly abundant and functionally important RNAs encompassing several groups involved in diverse cellular processes. Out of 3134 clusters fitting our inclusion criteria, 87 were located on gene-less scaffolds, whilst 1014 were located outside the candidate genic regions. These tag clusters constitute approximately one third of the total count. We postulate that these intergenic tag clusters may represent TSSs for several classes of non-coding RNA genes in *G. m. morsitans*. We refer to them as intergenic tag clusters. However, we are aware that the term ‘intergenic’ is loosely defined as the genome is yet to be fully assembled. In addition, manual refinement of the predicted gene models is on-going. In insects, non-coding RNAs appear to occur primarily in intergenic and intronic sequences and at intron-exon junctions. In addition they are significantly associated with genes encoding developmental regulators [46, 47]. Exploration of intergenic regions with TSS tag clusters may cast new insights into the role of non-coding genomic regions *Glossina* spp evolution.

### Functional roles under the control of narrow and broad promoters

In general, broad promoters have been associated with developmentally regulated genes whereas narrow promoters are associated with tissue specific expression [14]. However, in this study exceptions to this rule were observed. Genes involved in chitin metabolism were over-represented in all core promoter classes. This underscores their importance during development. Chitin metabolism is crucial to insect morphogenesis which primarily relies on the ability to remodel chitin-containing structures [48]. The presence of chitin metabolism genes in all promoter classes suggests members of this gene family in Tsetse are transcribed using both narrow and broad promoters.

The small GTPase binding activity constituted the bulk of GO annotations in the broad without peak category. GTPases are required for several developmental events such as organization of the actin cytoskeleton and signaling by c-Jun N-terminal kinase and p38 kinase cascades [49, 50]. They have also been shown to participate in dorsal closure of the *Drosophila* embryo [51]. Loss of the *Drosophila* larval GTPase Miro has been implicated in dysfunction of the axonal mitochondrial transport, leading to abnormal subcellular distribution of mitochondria in neurons and muscles [52]. The GTPase Cdc42 has recently been shown as a vital component during *Drosophila* embryonic development [53].

### Stress response controlled by broad promoters

The GO term heat shock binding activity is over-represented in the broad without peak promoters. In *Aedes aegypti* larvae and pupae, this protein family has been shown as an important indicator of stress and may function as crucial proteins to protect and improve survival [54]. During embryogenesis in *D.melanogaster*, expression of HSP60A is post-transcriptionally regulated in a highly dynamic order, even under heat-shock conditions suggesting novel roles for HSP60 family proteins throughout *Drosophila* development [55].

## Conclusions

By locating *G. m. morsitans* TSS using experimental data, the study has provided insight into the promoter architecture of *G. m. morsitans*. To our knowledge, this is the first study to locate TSSs and core promoters in the newly sequenced *G. m. morsitans* genome and by extension the first blood feeding vector. Results presented herein have generated testable hypothesis, such as the impact of transposons on transcription regulation. This study provided useful insights using *G. m. morsitans* genome data that would be employed as a platform to assess the conservation of transcriptional control mechanisms in upcoming *Glossina* genomes.

## Methods

### Acquisition of *G. m. morsitans* TSS seq data and preprocessing

TSS seq is a method that enables high throughput analysis of the mRNA sequence immediately downstream of the transcriptional start sites. This method replaces the cap structure of mRNA with the synthetic oligo, which contains the sequence adaptor sites, by enzymatic reactions [12]. *G. m. morsitans* RNA samples from pupae and larvae were prepared using the TRIzol protocol [56] at the Yale school of Public health and sent to the sequencing facility at the Genomic Sciences Center in Riken on dry ice. TSS seq was employed to produce sequence libraries using Illumina genome analyzer. The sequencing reactions were performed according to the manufacturer’s instructions. FASTQ files for these larval and pupal TSS seq reads were downloaded from the DNA data bank of Japan (DDBJ) in May 2012 [57] experiments SRX004541 and SRX004542 respectively. The files were combined to constitute one file containing approximately 17 million reads. The FASTX v 0.13 toolkit [58] was used for read preprocessing by first clipping of adapter sequences using the *fastx_clipper*. Trimming was performed using the *fastx_trimmer* with quality filtering by flagging –t for minimum quality threshold and -l for minimum length of read to be retained after trimming. Several rounds of trimming with variations in –l were performed to facilitate optimization of the mapping process. After trimming, the reads were processed with the *fastq_quality_filter* with –q and –p flags set at 31 and 50 respectively. For the reads that passed the quality filtering step, mapping was done using NOVOALIGN [59]. A summary of the read mapping statistics is presented in Additional file 5.

### Identification of TSS seq tag clusters

The methodology for assigning tag clusters is similar to that employed by Carninci et al. [28]. The primary difference relates to mapping of Illumina reads to the genome compared to CAGE tags (21 nucleotides). In the read alignment step, only reads that mapped uniquely were retained. The BEDTOOLS suite [60] together with custom scripts were used for the clustering process. Firstly, the alignment was converted from binary alignment (bam) format to browser extendible (bed) format after which the MERGEBED tool was used to merge overlapping reads into a single cluster with –d set to one so that all reads with at least one overlapping base were merged into one cluster. The –s option was flagged to force strandedness and –n to obtain a count of the reads that were contained in the corresponding clusters. Clusters that contained at least 100 TSS seq reads were selected and denoted as tag clusters. During our previous analysis [34] a minimum of 10 tags were used but with additional supporting experimental evidence such as full-length cDNAs. In our current study, ESTs with varying 5′ regions were available but these could not add accurate definition to the start of a gene. In the absence of supporting experimental data that could map to our Tag clusters, we required a higher number of tags to define the location of a tag clusters to eliminate false positives. We repeated the analysis with 50 tags and obtained similar promoter patterns (Additional files 6 and 7). The CLOSESTBED tool was used to identify classify tag cluster position relative to the 5′UTR. Classification of tag clusters mapping onto the 5′UTR was done as follows;

(i) *Bona fide* 5′UTR clusters = tag clusters mapping onto 5′UTR regions.
(ii) Other 5′UTR clusters = tag clusters mapping to genes without a demarcated 5′UTR region.

Tag cluster(s) were retained if they mapped within 300 bases (mean 5′ UTR length) upstream from the start of the first coding exon. In addition, genes with an extremely short (1–10 bases) 5′UTRs but with a tag cluster(s) mapped within 300 bases (mean 5′ UTR length) upstream from the start of the 5′UTR were also included. We did not exclude the tag clusters that mapped in the first coding exons because it has been shown that the locations of TSS fluctuate to some extent in most genes [28, 61, 62].

### Identification of dominant TSS and core promoter extraction

A TSS position was defined as the position with the highest frequency of tag counts for each delineated cluster. A custom script was used to extract and calculate the frequency of read start positions for each cluster. A comprehensive illustration of the TSS and promoter identification pipeline is attached in Additional file 8. The scripts used in these analyses are available on the South African National Bioinformatics Institute permanent data archive (ftp://ftp.sanbi.ac.za/Glossina_TSS).

### Delineation of promoter classes

Core promoters were defined as one hundred bases in the milieu of the TSS that is the −50/+50 positions relative to the TSS (+1). Some broad core promoters exhibit properties of both narrow and broad initiation patterns where they exhibit propensity for one TSS. These are classified as “broad with peak” while those that do not exhibit propensity for one particular TSS are referred to as “broad without peak” [63]. To delineate the shapes (with or without peak) of a tag cluster, we employed the individual peakedness score method as described by Zhao and colleagues [64]. This method evaluates the peakedness of tag clusters by defining the individual peakedness “*s”* of a tag cluster “*g*” by the formula:

$$ {S}_g=\frac{m}{nw} $$

Where *m* is the tag count at the dominant peak (the mode) *n* is the total number of reads in the distribution, *w* is the width of the distribution, that is, the genomic window covered by the tag cluster. Accordingly, each cluster had a discrete individual peakedness score. Notably the maximum individual peakedness score had value of 1, where a cluster has only one TSS position. The higher the individual peakedness score, the more defined the TSS genomic location. An illustration of an implementation of this formula is shown in Additional file 9.

The absolute count of TSS positions in a given tag cluster was used to classify broad versus narrow promoters. All clusters with 10 or less TSS positions were classified as narrow whilst the remainders were classified as broad. Broad clusters were further categorized based on their peakedness. A broad cluster was deemed as one with a dominant peak if the TSS with the highest frequency of tag counts constituted at least 50 % of the total tag count. If this condition was not met, the broad cluster was classified as “without a peak” (see Additional file 10 for an illustration).

### Core promoter extraction

Core promoters were defined as 100 nucleotides (−50/+50) TSS surrounding the TSS. An in house script was used to extract the core promoters. For candidate regions with more than one tag cluster, the cluster with the highest number of TSS seq tags was selected for further analysis. Here, the TSS position with the highest number of TSS seq tags was selected for core promoter extraction. To avoid confounding results during motif search, core promoters entangled with transposons were eliminated.

### Nucleotide composition analysis

To compare the nucleotide composition between mammalian and insect genomes, human and *Drosophila* promoter datasets utilized in the study by FitzGerald and colleagues [19] were obtained. The mouse promoter dataset was obtained from the Eukaryotic promoter database [65]. Regions spanning the TSS from −200 to +100 positions were extracted and aligned to assess percent nucleotide composition at each position using the FASTX toolkit [58].

### Annotation of core promoter motifs

In the absence of experimentally verified core promoter motifs for *Glossina morsitans* hitherto, core promoter associated PWMs were downloaded from the JASPAR database [66]. MATRIX-SCAN program [67] of the RSAT suite [68] was used to scan core promoter sequences. The p-value threshold was set at 1^e-02^ to distinguish true positives from false positives and the program was set to estimate a background model from the input sequence. Only those core promoter motifs occurring at their corresponding biologically functional genomic windows were captured. Because core promoter motif placement often exhibits elasticity [2], the canonical start positions were allowed to vary by +/−5 bp within their corresponding genomic windows for which they are biologically functional (see Additional file 11 for tabulation of the genomic windows). Because TFBSs are typically short (5–15 nucleotides) and tolerate generally high levels of sequence degeneracy, majority of common motif finding algorithms may not accurately discriminate *bona fide* motifs from remaining sequence. This phenomenon has been described as the motif “twilight zone” where every motif finding algorithm is ordinarily exposed to spurious matches that may appear as significant as the ones in question [24]. By knowing the biologically functional genomic windows of these motifs a priori, motifs that did not occur in biologically functional genomic windows were excluded, partly correcting for spurious matches. Further, random promoter sets for each of the promoter classification (narrow, broad with peak and broad without peak) were generated by shuffling the core promoter sequences. A similar technique was employed by Frith et al. [69] and Jin et al. [5]. Though non-functional, the random promoter sets have the same nucleotide frequencies as the true core promoter sequences. Accordingly, they are preferable to coding or inter-genic sequences, which exhibit nucleotide bias. The core promoter motifs annotation procedure was repeated for the random promoter datasets. A paired binomial test was performed to determine whether there were significant differences in the numbers of core promoters harboring motifs at biologically functional genomic windows between the *bona fide* and randomly generated core promoter datasets. Motif co-occurrence suggests combinatorial regulation of transcription via physical interactions between corresponding TFs. To elucidate patterns of motif co-occurrence in various core promoter classes, two-way and three-way motif co-occurrences were evaluated for each core promoter sequence. Further, core promoter sequences with a TATA in a biologically functional window (TATA-containing) were separated from core promoters without a TATA in a biologically functional window (TATA-less). The frequency of motifs in TATA-containing versus TATA-less categories was also evaluated. A summary of the methodology implemented for annotation of core promoter motifs is shown in Additional file 12.

### Motif co-occurrence analysis

Essentially, positive associations between motifs suggest likelihood of physical interactions between the TFs that bind the co-occurring motifs. On the other hand, negative correlations imply that the TFs that bind them have divergent functions. The two-way and three-way motif co-occurrence was computed for each core promoter class producing fifteen and twenty possible combinations respectively. For each core promoter category, the combination with the highest percent co-occurrence was identified. To evaluate for over-representation of the various combinations, a 95 % confidence interval of each combination with respect to the sample size was calculated.

### Gene ontologies associated with various promoter classes

The ability of TSS seq to facilitate digital expression profiling was exploited. Since larvae and pupae samples were used to create TSS-seq libraries, it was hypothesized that gene products characteristic of development would be over-represented. To evaluate for overrepresentation of certain biological/molecular processes in the three promoter classes, Gene Ontology (GO) [70] annotations were obtained for every gene in each promoter classification. For each promoter class, GO annotations were summed and the summary statistics computed. Annotations falling within the 75th percentile of their corresponding summary statistics were deemed as over-represented. Additional file 13 is a summary illustration of the methodology employed for GO analysis.

### Ethics

No ethical approval was needed for this study.

## Availability of supporting data

The scripts used in these analyses are available on the South African National Bioinformatics Institute permanent data archive (ftp://ftp.sanbi.ac.za/Glossina_TSS).

FASTQ files for larval and pupal TSS seq reads were downloaded from the DNA data bank of Japan (DDBJ) [57] experiments SRX004541 and SRX004542 respectively. All other supporting data are included as additional files.

## Additional files

Additional file 1:
**Ontology terms occurring in the 75th percentile of narrow core promoters.** In the narrow category, the ontology terms structural constituent of cuticle, ATP binding and DNA binding recorded highest frequency. (PNG 73 kb) Additional file 2:
**Ontology terms occurring in the 75th percentile of broad with peak core promoters.** In the broad with peak category, the ontology terms structural constituent of cuticle, oxidoreductase with molecular oxygen activity and structural constituent of ribosome recorded highest frequency. (PNG 120 kb) Additional file 3:
**Ontology terms occurring in the 75th percentile of broad with peak core promoters.** In the broad with peak category, the ontology terms structural constituent of cuticle, oxidoreductase with molecular oxygen activity and structural constituent of ribosome recorded highest frequency. (PNG 214 kb) Additional file 4:
**Percent occurrence of core promoter motifs in various core promoter classes.** (DOC 26 kb) Additional file 5:
**Summary of read mapping statistics.** (DOC 28 kb) Additional file 6:
**Two-way motif co-occurrences at 50 tags per cluster cut-off.** (DOC 40 kb) Additional file 7:
**Three-way motif co-occurrences at 50 tags per cluster cut-off.** (DOC 46 kb) Additional file 8:
**A comprehensive illustration of the TSS and promoter identification pipeline.** (PNG 184 kb) Additional file 9:
**Histogram of a sample TSS-seq tag cluster.** The x axis represents the genomic TSS positions where TSSseq reads map (the corresponding scaffold ID is denoted) while the y axis represents the frequency of reads at each TSS. Substituting the values according to the equation above; The mode (m) / TSS position with highest number of reads =107. There are 49 TSS positions, thus the width (w) = 49. The sum (n) of the reads (counts) is: (9+10+1+50+1+9+107+1+2+1+3+1+5+21+1+2+1+1+1+1+1+5+1+3+1+3+1+4+1+6+1+3+1+3+2+11+1+4+11+1+1+7+20+1+3+1+2+47+1) = 375. The individual peakedness score will be 107 ÷ (375*49) = 0.005823129. (PNG 165 kb) Additional file 10:
**Graphical impressions of representative tag clusters for the various promoter classes.** The x-axis represents the genomic position. The corresponding scaffold ID is denoted. The y-axis represents the percent of the total tag count at each genomic position. Figure (a) is a representative of the narrow class whose TSS positions span five nucleotides with a single dominant peak. Figures b and c denote the broad promoter classes whose TSS spans several to hundreds of nucleotides. This class can either be broad with a dominant peak (b) where the dominant peak constitutes approximately 80 % of the total tag count or broad with multiple but no dominant peaks (c) where there is no single dominant peak. (PNG 285 kb) Additional file 11:
**Core promoter genomic windows used for the analysis.** (DOC 29 kb) Additional file 12:
**A comprehensive illustration showing the methodology implemented for annotation of core promoter motifs.** (PNG 243 kb) Additional file 13:
**Summary of core promoter GO annotations analysis methodology.** (PNG 167 kb)

## Abbreviations

BREd: Downstream TFIIB recognition element
BREu: Upstream TFIIB recognition element
CDS1: First coding exon
DDBJ: DNA data bank of Japan
*D melanogaster*: *Drosophila melanogaster*
DNA: Deoxyribonucleic acid
DPE: Downstream promoter element
*G. m. morsitans*: *Glossina morsitans morsitans*
INR: Initiator
MTE: Motif ten element
NGS: Next generation sequencing
PWM: Position weight matrix
RNA: Ribonucleic acid
TF: Transcription factor
TFBS: Transcription factor binding site
TSS: Transcription start site
UTR: Untranslated region
