# Supplementary figures and images for: TSS seq based core promoter architecture in blood feeding Tsetse fly (Glossina morsitans morsitans) vector of Trypanosomiasis
Source: BMC Genomics. 2015 Sep 22;16(1):722. doi: 10.1186/s12864-015-1921-6 (PMC4578606; doi:10.1186/s12864-015-1921-6)

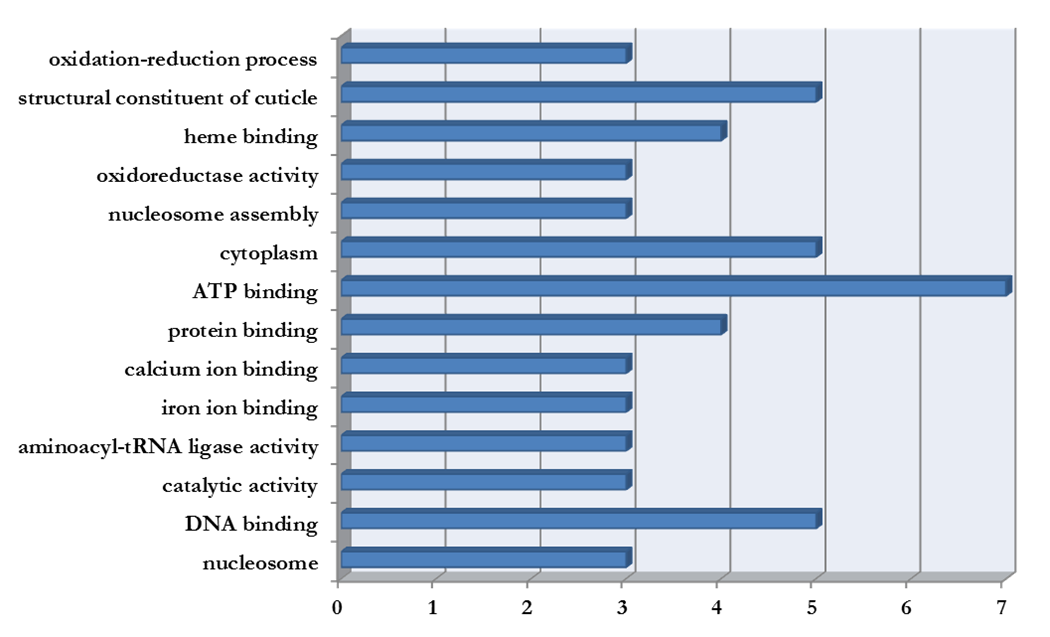

Supplement: Additional file 1: — Ontology terms occurring in the 75th percentile of narrow core promoters. In the narrow category, the ontology terms structural constituent of cuticle, ATP binding and DNA binding recorded highest frequency. (PNG 73 kb) [file 12864_2015_1921_MOESM1_ESM.png]

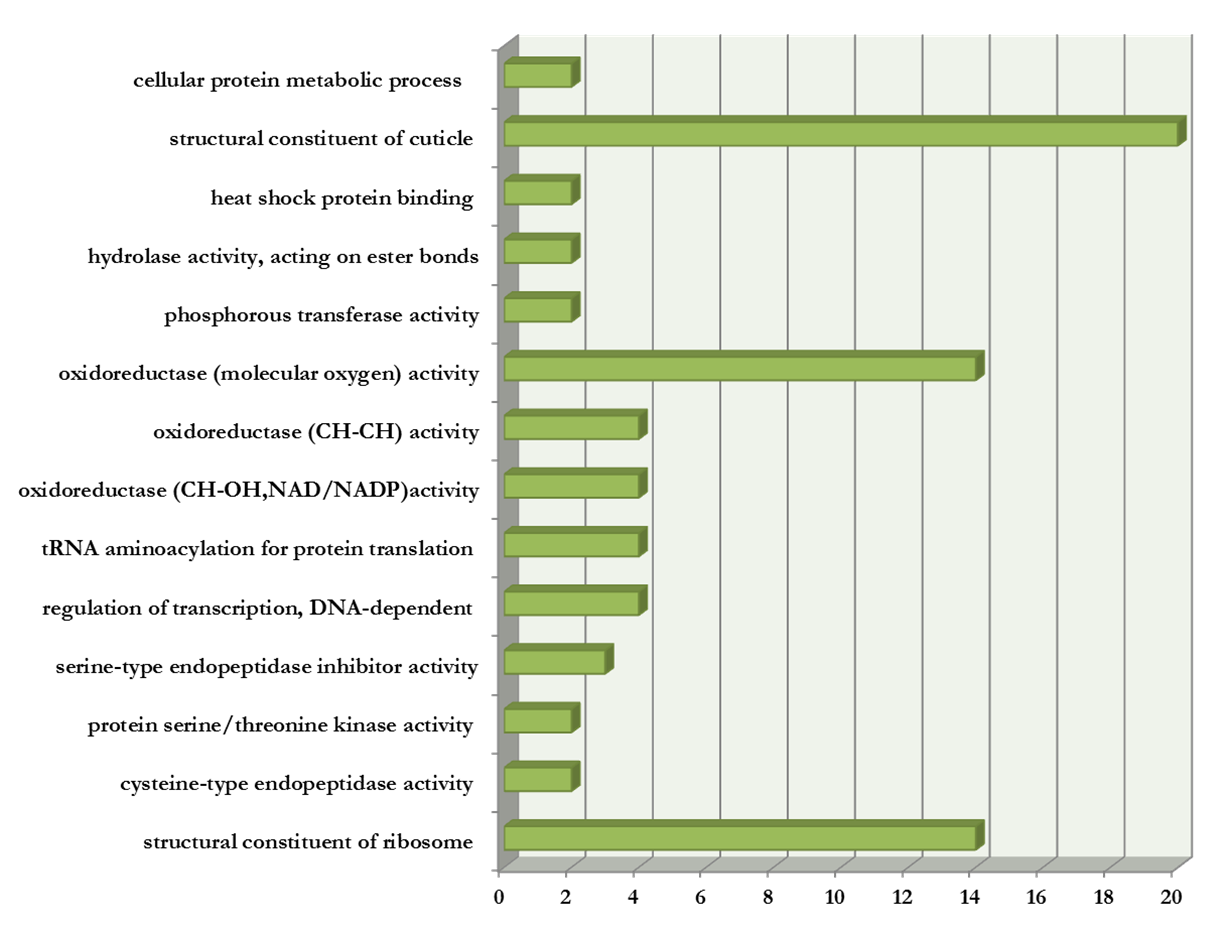

Supplement: Additional file 2: — Ontology terms occurring in the 75th percentile of broad with peak core promoters. In the broad with peak category, the ontology terms structural constituent of cuticle, oxidoreductase with molecular oxygen activity and structural constituent of ribosome recorded highest frequency. (PNG 120 kb) [file 12864_2015_1921_MOESM2_ESM.png]

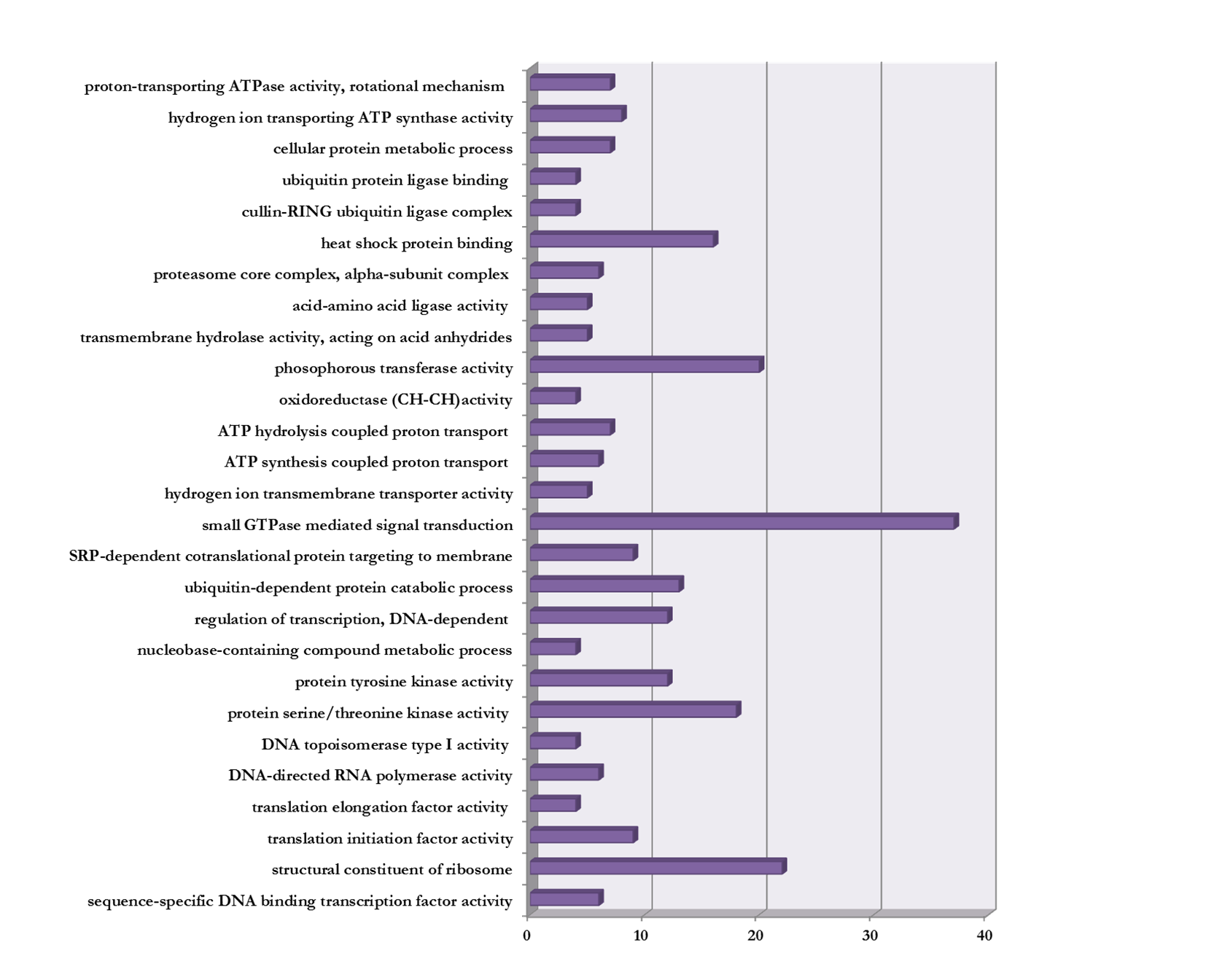

Supplement: Additional file 3: — Ontology terms occurring in the 75th percentile of broad with peak core promoters. In the broad with peak category, the ontology terms structural constituent of cuticle, oxidoreductase with molecular oxygen activity and structural constituent of ribosome recorded highest frequency. (PNG 214 kb) [file 12864_2015_1921_MOESM3_ESM.png]

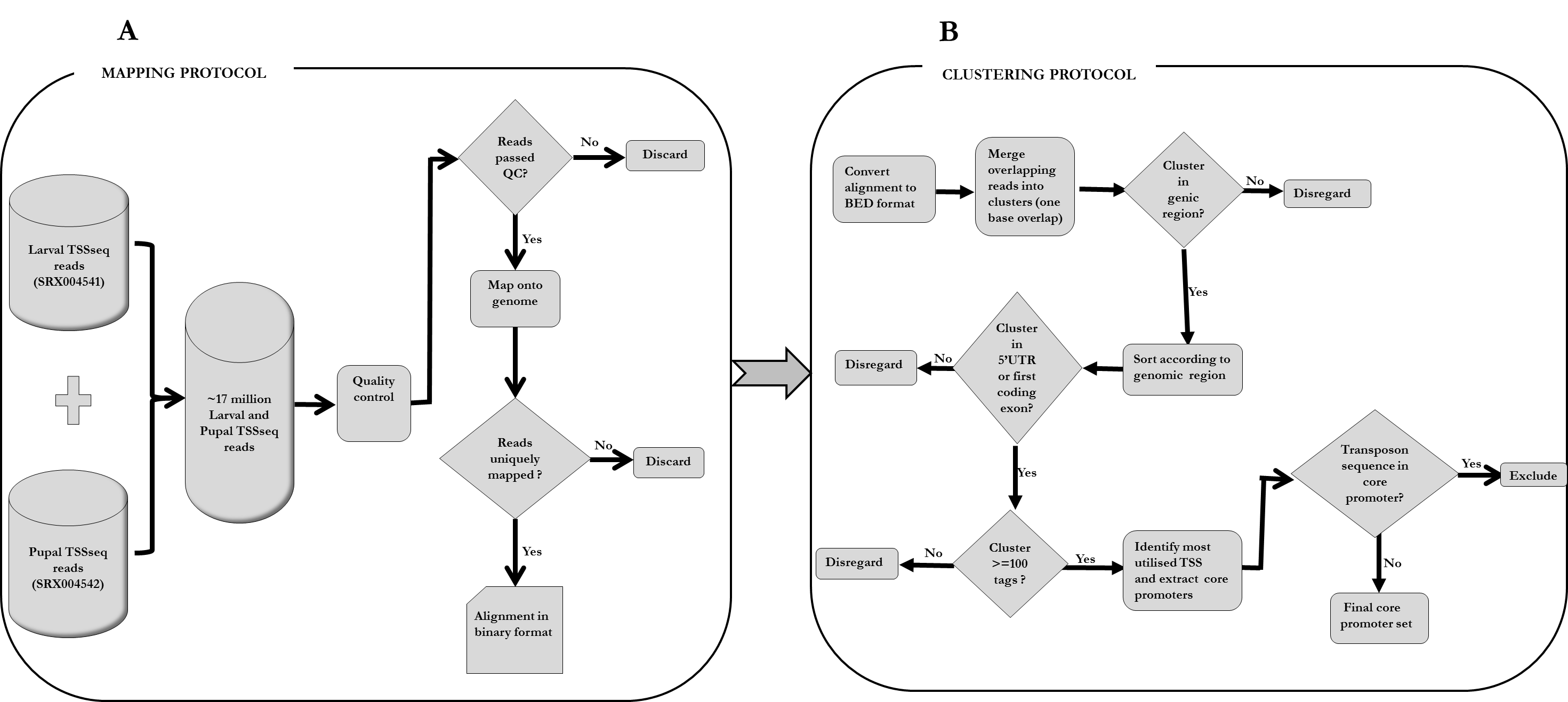

Supplement: Additional file 8: — A comprehensive illustration of the TSS and promoter identification pipeline. (PNG 184 kb) [file 12864_2015_1921_MOESM8_ESM.png]

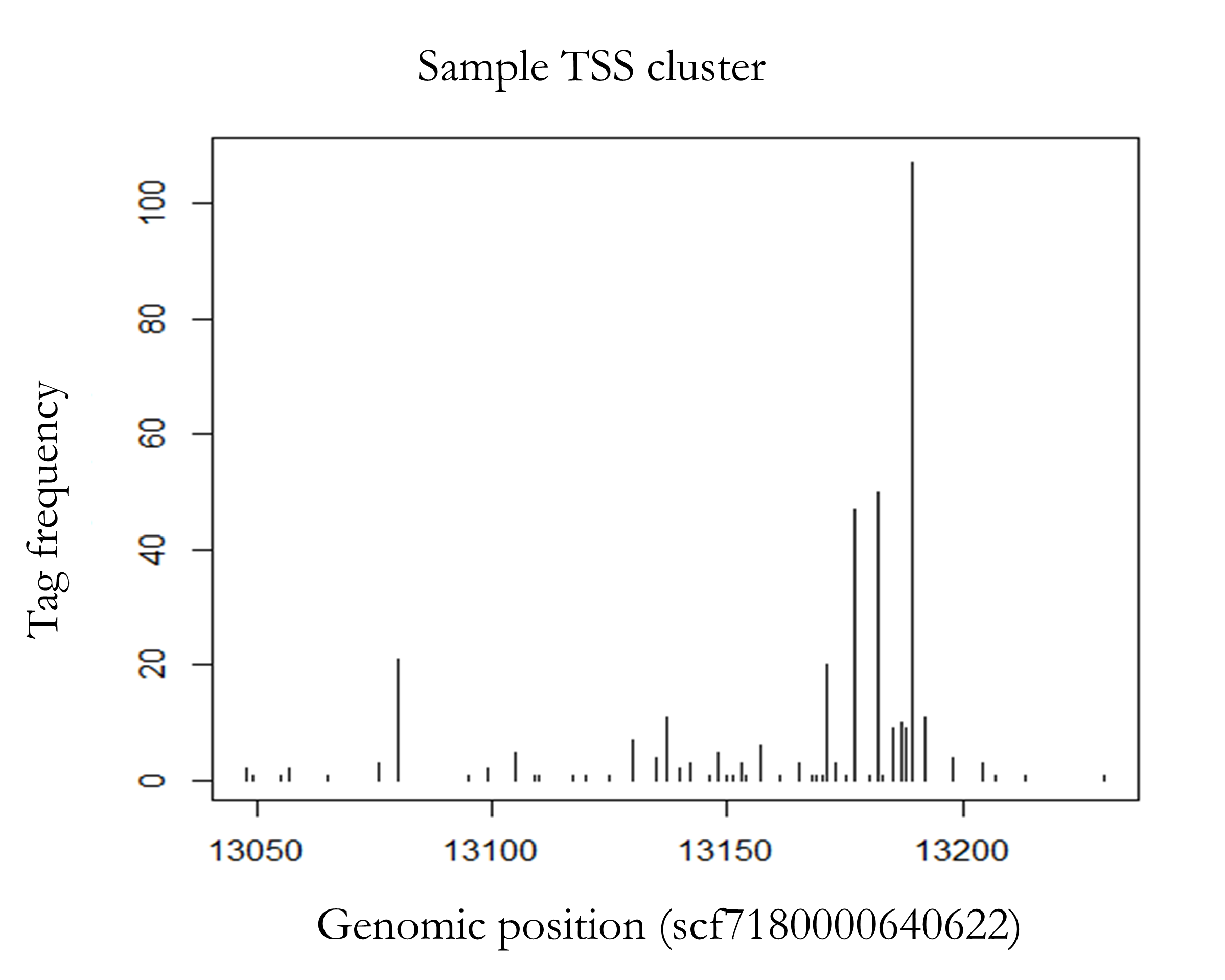

Supplement: Additional file 9: — Histogram of a sample TSS-seq tag cluster. The x axis represents the genomic TSS positions where TSSseq reads map (the corresponding scaffold ID is denoted) while the y axis represents the frequency of reads at each TSS. Substituting the values according to the equation above; The mode (m) / TSS position with highest number of reads =107. There are 49 TSS positions, thus the width (w) = 49. The sum (n) of the reads (counts) is: (9+10+1+50+1+9+107+1+2+1+3+1+5+21+1+2+1+1+1+1+1+5+1+3+1+3+1+4+1+6+1+3+1+3+2+11+1+4+11+1+1+7+20+1+3+1+2+47+1) = 375. The individual peakedness score will be 107 ÷ (375*49) = 0.005823129. (PNG 165 kb) [file 12864_2015_1921_MOESM9_ESM.png]

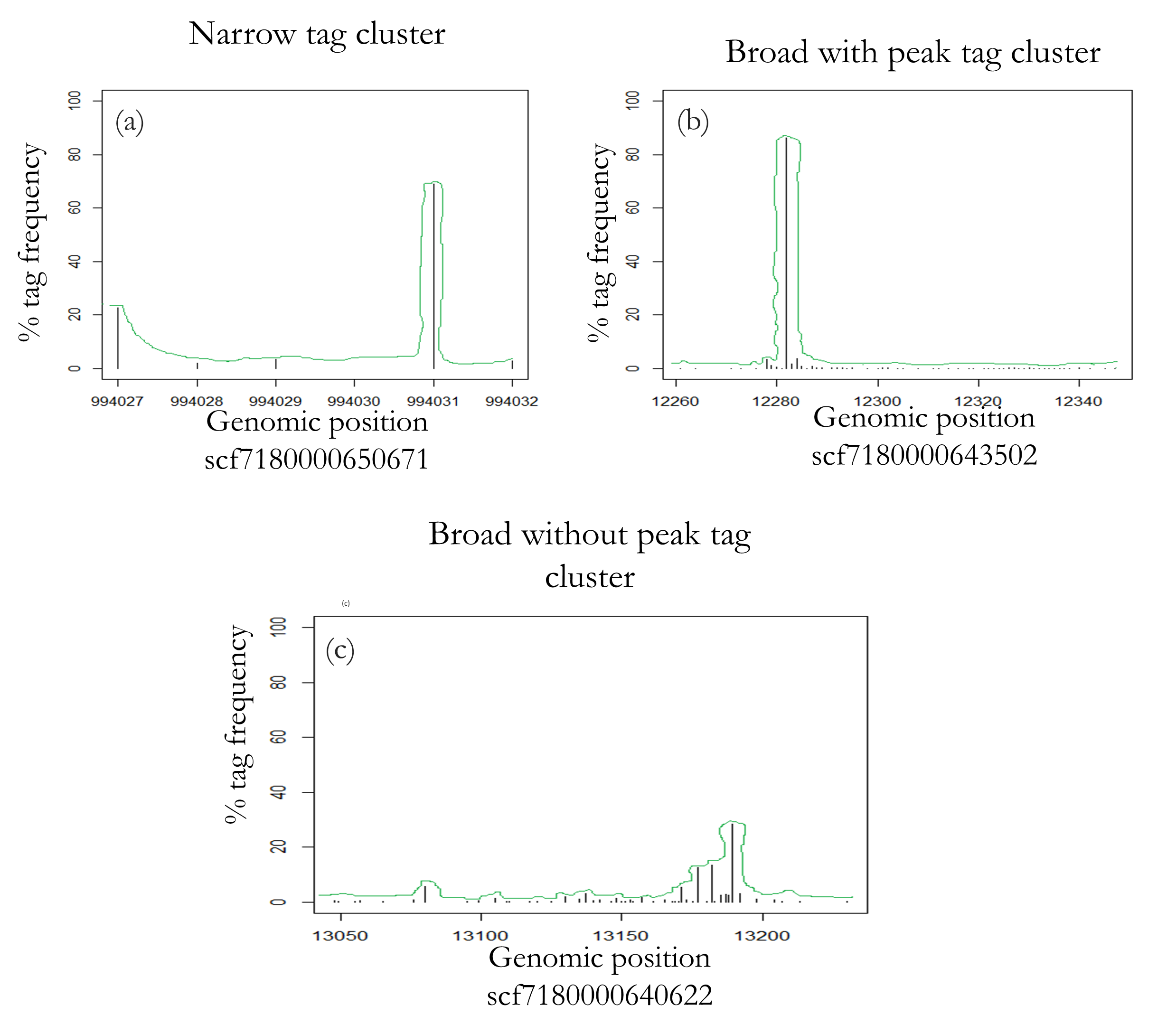

Supplement: Additional file 10: — Graphical impressions of representative tag clusters for the various promoter classes. The x-axis represents the genomic position. The corresponding scaffold ID is denoted. The y-axis represents the percent of the total tag count at each genomic position. Figure (a) is a representative of the narrow class whose TSS positions span five nucleotides with a single dominant peak. Figures b and c denote the broad promoter classes whose TSS spans several to hundreds of nucleotides. This class can either be broad with a dominant peak (b) where the dominant peak constitutes approximately 80 % of the total tag count or broad with multiple but no dominant peaks (c) where there is no single dominant peak. (PNG 285 kb) [file 12864_2015_1921_MOESM10_ESM.png]

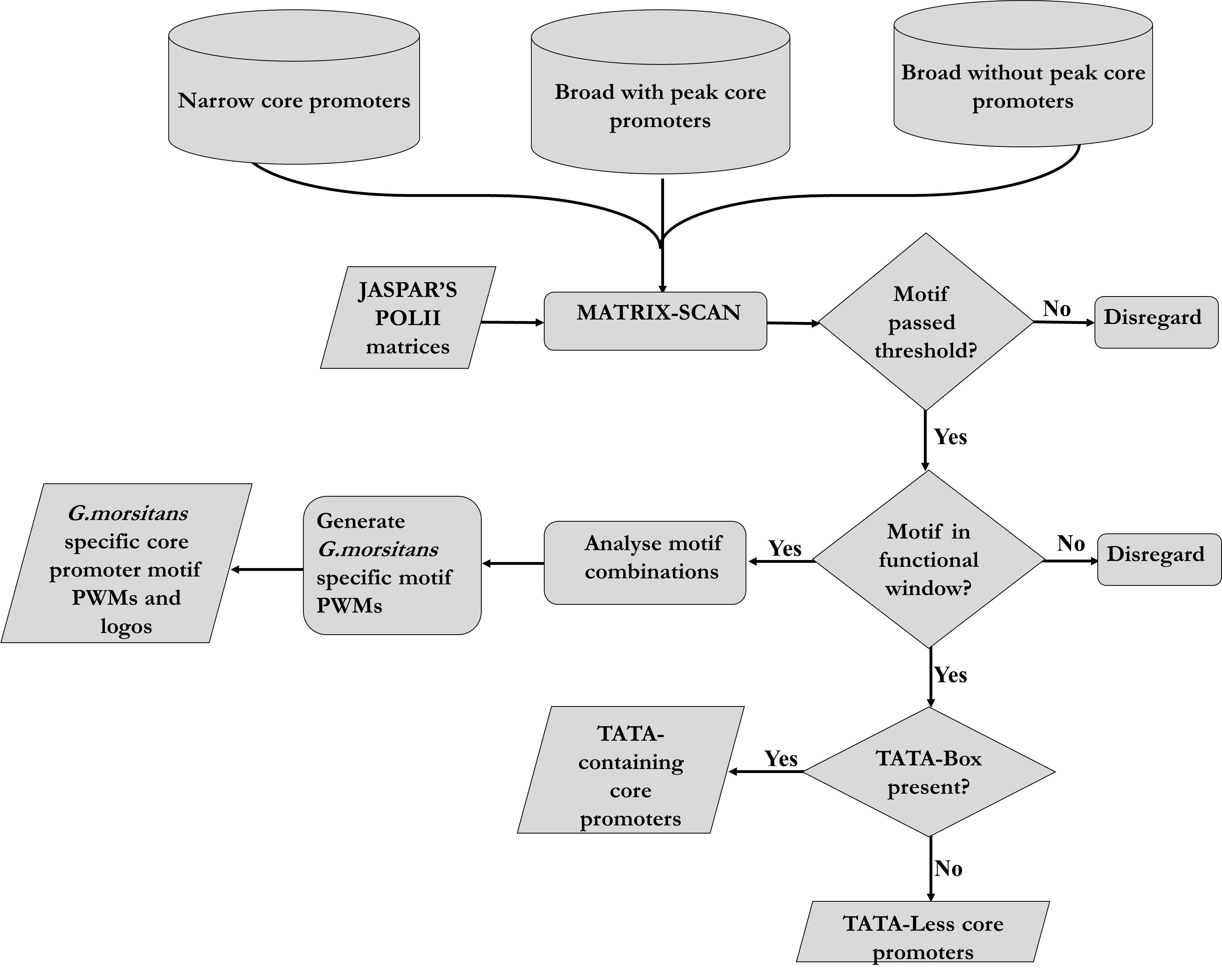

Supplement: Additional file 12: — A comprehensive illustration showing the methodology implemented for annotation of core promoter motifs. (PNG 243 kb) [file 12864_2015_1921_MOESM12_ESM.png]

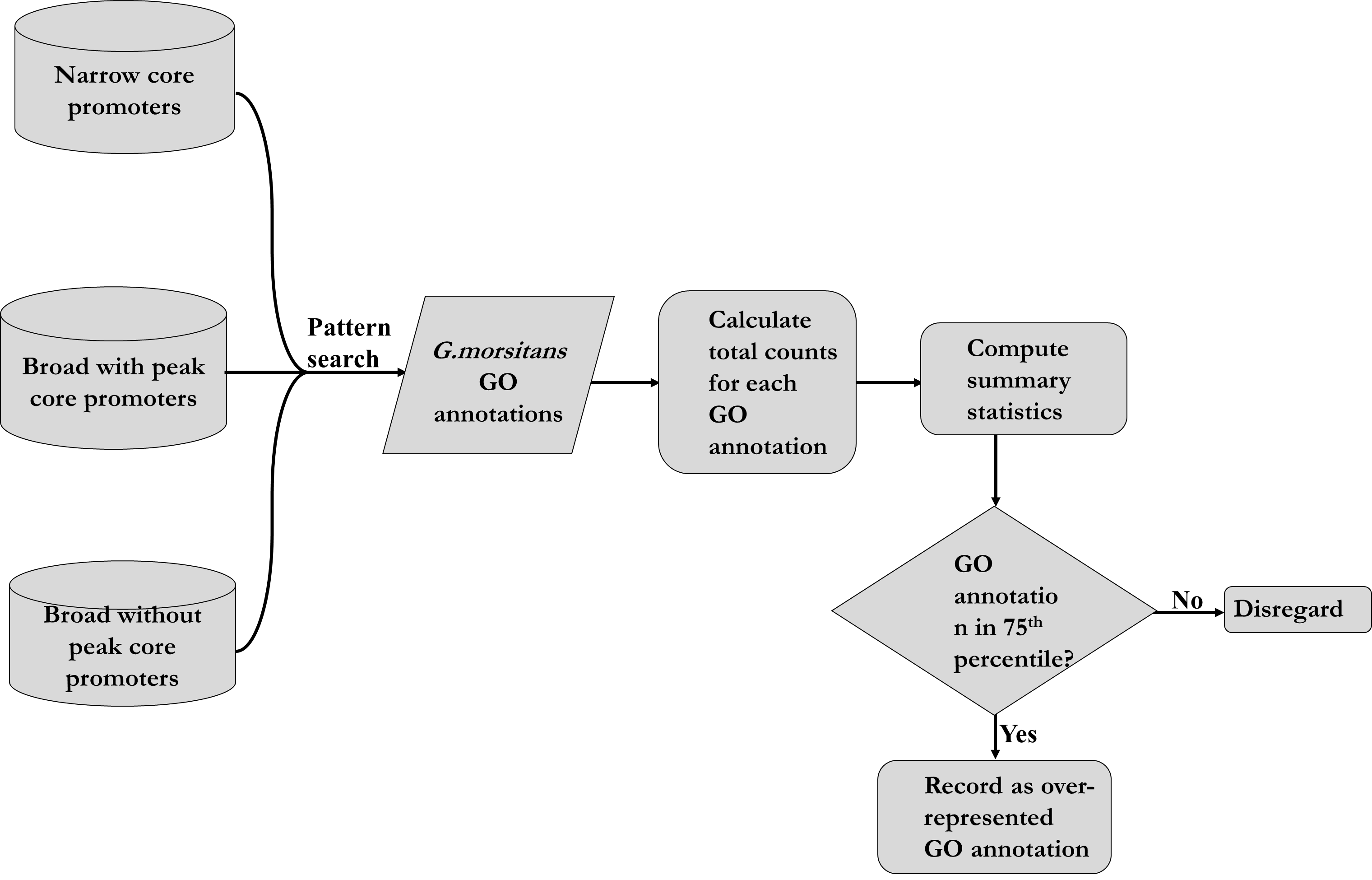

Supplement: Additional file 13: — Summary of core promoter GO annotations analysis methodology. (PNG 167 kb) [file 12864_2015_1921_MOESM13_ESM.png]
