# Supplementary material for: TSS seq based core promoter architecture in blood feeding Tsetse fly (Glossina morsitans morsitans) vector of Trypanosomiasis
Source: BMC Genomics. 2015 Sep 22;16(1):722. doi: 10.1186/s12864-015-1921-6 (PMC4578606; doi:10.1186/s12864-015-1921-6)
Supplement: Additional file 4: — Percent occurrence of core promoter motifs in various core promoter classes. (DOC 26 kb) [file 12864_2015_1921_MOESM4_ESM.doc]

Additional file 4: Percent occurrence of core promoter motifs in various core promoter classes.

| % Occurrence | | | | | | |
| --- | --- | --- | --- | --- | --- | --- |
| Core promoter classes | BREu | BREd | TATA | INR | MTE | DPE |
| Narrow | 27 | 24 | 49 | 51 | 36 | 29 |
| Broad with peak | 18 | 28 | 41 | 40 | 34 | 37 |
| Broad without peak | 21 | 30 | 27 | 30 | 27 | 31 |
