# Supplementary material for: TSS seq based core promoter architecture in blood feeding Tsetse fly (Glossina morsitans morsitans) vector of Trypanosomiasis
Source: BMC Genomics. 2015 Sep 22;16(1):722. doi: 10.1186/s12864-015-1921-6 (PMC4578606; doi:10.1186/s12864-015-1921-6)
Supplement: Additional file 5: — Summary of read mapping statistics. (DOC 28 kb) [file 12864_2015_1921_MOESM5_ESM.doc]

Additional file 5: Summary of read mapping statistics

| **Parameter** | **Count** |
| --- | --- |
| Total number of reads from DDBJ | 17,218,719 |
| Reads that passed the quality filtering step | 10,543,105 |
| Reads that reported at least one alignment | 7,048,660 |
| Reads that reported unique alignment | 6,622,424 |
