# Supplementary material for: TSS seq based core promoter architecture in blood feeding Tsetse fly (Glossina morsitans morsitans) vector of Trypanosomiasis
Source: BMC Genomics. 2015 Sep 22;16(1):722. doi: 10.1186/s12864-015-1921-6 (PMC4578606; doi:10.1186/s12864-015-1921-6)
Supplement: Additional file 6: — Two-way motif co-occurrences at 50 tags per cluster cut-off. (DOC 40 kb) [file 12864_2015_1921_MOESM6_ESM.doc]

Additional file 6: Two-way motif co-occurrences at (50 tags per cluster cut-off)

|  | | **% Co-occurrence in core promoter category** | | |
| --- | --- | --- | --- | --- |
| Motif combination | Narrow | | Broad with peak | Broad without peak |
| BREu-TATA | 12 | | 10 | 12 |
| BREu-BREd | 10 | | 16 | 10 |
| BREu-INR | 11 | | 12 | 9 |
| BREu-MTE | 9 | | 6 | 10 |
| BREu-DPE | 16 | | 12 | 7 |
| TATA-BREd | 14 | | 14 | 12 |
| TATA-INR* | 31 | | 17 | 16 |
| TATA-MTE | 26 | | 12 | 14 |
| TATA-DPE | 19 | | 18 | 12 |
| BREd-INR | 14 | | 10 | 9 |
| BREd-MTE | 14 | | 12 | 9 |
| BREd-DPE | 9 | | 11 | 12 |
| INR-MTE* | 33 | | 13 | 15 |
| INR-DPE | 18 | | 18 | 15 |
| MTE-DPE** | 16 | | 29 | 21 |

*High frequency of occurrence in narrow core promoters.

** Statistically over represented in both broad with peak and broad without peak core promoters
