# Supplementary material for: TSS seq based core promoter architecture in blood feeding Tsetse fly (Glossina morsitans morsitans) vector of Trypanosomiasis
Source: BMC Genomics. 2015 Sep 22;16(1):722. doi: 10.1186/s12864-015-1921-6 (PMC4578606; doi:10.1186/s12864-015-1921-6)
Supplement: Additional file 7: — Three-way motif co-occurrences at 50 tags per cluster cut-off. (DOC 46 kb) [file 12864_2015_1921_MOESM7_ESM.doc]

Additional file 7: Three-way motif co-occurrences at (50 tags per cluster cut-off)

|  | **% Co-occurrence in core promoter** | | |
| --- | --- | --- | --- |
| Motif combination | Narrow | Broad with peak | Broad without peak |
| BREu-TATA-BREd | 26 | 24 | 20 |
| BREu-TATA-INR | 38 | 24 | 25 |
| BREu-TATA-MTE | 32 | 18 | 22 |
| BREu-TATA-DPE | 29 | 25 | 21 |
| BREu-BREd-INR | 22 | 22 | 19 |
| BREu-BREd-MTE | 25 | 21 | 18 |
| BREu-BREd-DPE | 21 | 22 | 19 |
| BREu-INR-MTE | 39 | 22 | 22 |
| BREu-INR-DPE | 29 | 26 | 21 |
| BREu-MTE-DPE | 25 | 31 | 25 |
| TATA-BREd-INR | 43 | 27 | 24 |
| TATA-BREd-MTE | 35 | 22 | 23 |
| TATA-BREd-DPE | 30 | 27 | 23 |
| TATA-INR-MTE | 50 | 27 | 28 |
| TATA-INR-DPE* | 45 | 32 | 26 |
| TATA-MTE-DPE* | 44 | 33 | 28 |
| BREd-INR-MTE | 39 | 23 | 22 |
| BREd-INR-DPE | 27 | 25 | 23 |
| BREd-MTE-DPE | 29 | 35 | 29 |
| INR-MTE-DPE* | 42 | 38 | 29 |

*High frequency of occurrence in all core promoter categories.
