# Supplementary material for: TSS seq based core promoter architecture in blood feeding Tsetse fly (Glossina morsitans morsitans) vector of Trypanosomiasis
Source: BMC Genomics. 2015 Sep 22;16(1):722. doi: 10.1186/s12864-015-1921-6 (PMC4578606; doi:10.1186/s12864-015-1921-6)
Supplement: Additional file 11: — Core promoter genomic windows used for the analysis. (DOC 29 kb) [file 12864_2015_1921_MOESM11_ESM.doc]

Additional file 11: Core promoter genomic windows used for the analysis

| **Core promoter**  **motif** | **Genomic window**  **for motif start** |
| --- | --- |
| BREu | -37 to -27 |
| TATA-box | -31 to -21 |
| BREd | -23 to -13 |
| INR | -5 to +5 |
| MTE | +18 to+28 |
| DPE | +28 to +38 |
